# Evaluation of fluoride varnish treatment of postorthodontic white spot lesions by visual inspection and laser fluorescence—A randomized controlled study

**DOI:** 10.1002/cre2.579

**Published:** 2022-05-05

**Authors:** Tanja Tomaževič, Martina Drevenšek, Rok Kosem

**Affiliations:** ^1^ Department of Paediatric and Preventive Dentistry University Medical Centre Ljubljana Ljubljana Slovenia; ^2^ Department of Orthodontics, Faculty of Medicine, University of Ljubljana University Medical Centre Ljubljana Ljubljana Slovenia; ^3^ Department of Paediatric and Preventive Dentistry University Medical Centre Ljubljana Ljubljana Slovenia

**Keywords:** fluoride varnish, postorthodontic, white spot lesion

## Abstract

**Objectives:**

White spot lesions (WSLs), as a side effect of orthodontic therapy, can be treated with fluoride varnish, with the difference in efficiency reported.

**Material and methods:**

Patients with buccal WSLs were consecutively included in a randomized controlled double‐blind study. At first inspection and at three follow‐ups over 6 months, 0.1% fluoride varnish and placebo (water) were applied in the test group (*N* = 21) and control group (*N* = 21), respectively. The maximum laser fluorescence value (LFV) of WSLs was recorded using DIAGNOdent. Between the groups, differences in the mean numbers of WSLs and the mean LFV of WSLs per patient at different time points were analyzed with mixed‐design analysis of variance. Orthodontic therapy duration (OTD) was included in the model as a covariate.

**Results:**

A decrease in the mean WSLs number and LFV was observed; however, there were no significant differences between study groups at any time point. OTD was in interaction only with LFV. Analysis showed a different pattern of mean LFV changes for patients with OTD of >48 months compared to patients with OTD of ≤24.

**Conclusion:**

The changes in numbers of WSLs and LFV over the study period indicated regression of WSLs, but an additional effect of FV was not confirmed.

## 1 INTRODUCTION

White spot lesions (WSLs) as an indication of enamel demineralization due to prolonged plaque accumulation are often seen during and after treatment with a fixed orthodontic appliance (FOA). In a literature review, Willmot (2008) summarized that the prevalence of WSL affected tooth surfaces in patients with FOA was from 4.9% to 84%. A high prevalence of WSL‐affected patients (97%) was determined by quantitative light fluorescence (Boersma et al., 2005). Good oral hygiene, aiming to remove dental plaque, and the application of topical remineralization agents are fundamental measures for reducing enamel demineralization during orthodontic therapy (Benson et al., 2013; Migliorati et al., 2015). After removing the FOA, enamel demineralization usually stops, due to the mineralizing effect of saliva and additional treatment protocols (Chen et al., 2013; Fujikawa et al., 2008; Ogaard & Ten Bosch, 1994). A positive effect of fluoride varnish (FV), (Du et al., 2012; He et al., 2016) fluoride film, (He et al., 2016) fluoride toothpaste, (Mensinkai et al., 2012) casein phosphopeptide‐amorphous calcium fluoride phosphate remineralizing crème (Llena et al., 2015), and fluoridated chewing sticks (Baeshen et al., 2011) has been shown in reducing demineralization and promoting WSL regression after orthodontic treatment. On the contrary, a systematic review showed that more reliable scientific evidence to support remineralizing strategies, among them the usage of FV in postorthodontic WSLs is needed (Sonesson et al., 2017). A certain number of WSLs will never regress (Mattousch et al., 2007) since porosity of the subsurface area may still be present due to the incorporation of ions in deeper WSL layers being prevented once the surface of the lesion has remineralized (Willmot, 2008).

The presence of a WSL is conventionally well detected by visual inspection, (Gimenez et al., 2015) but with a limited ability to assess the degree of demineralization and the de‐ and remineralization dynamics (Makhija et al., 2017). Laser fluorescence (LF) detecting devices have been used as an additional diagnostic method to monitor the dynamics of WSL de‐ and remineralization in a bacteria inhabited environment, as was shown in vitro and in situ by Spiguel et al. (2009) and in vivo by Ferreira et al. (2008). The results of a study by Aljehani et al. (2006) showed that the LF detecting device DIAGNOdent was able to detect remineralization of WSLs in postorthodontic patients, while it could not be detected visually over a 1‐year period.

Due to contradictory reports on FV effectiveness on regression of WSLs in postorthodontic patients and the concern that higher concentrations of fluoride arrest remineralization of deeper lesion layers because of surface hypermineralization, the primary aim of our study was to assess the effect of topical fluoride agent in the form of a 0.1% varnish on postorthodontic WSL regression in addition to the advocated regular use of fluoridated toothpaste. To the best of our knowledge, the effectiveness of 0.1% FV in postorthodontic patients has not been investigated yet. Additionally to visual inspection, LF measurement of WSLs using DIAGNOdent was performed. The hypothesis that we tested was that 0.1% FV in combination with regular usage of fluoride toothpaste is more effective in postorthodontic WSL regression than the usage of fluoridated toothpaste alone. A secondary aim was to investigate whether the duration of orthodontic treatment affects WSL regression.

## 2 STUDY POPULATION AND METHODOLOGY

This randomized double‐blind controlled study was conducted at the Department of Paediatric and Preventive Dentistry (DPPD) and Department of Orthodontics (DO).

### 2.1 Patients and eligibility criteria

The patients were consecutively recruited at DO and no special selection besides the eligibility criteria was employed for the purpose of this study. Prior to the enrollment, all patients or parents/legal guardians of patients younger than 18 years were informed verbally and by informed letter in lay language about the study aim and the study protocol. Written informed consent was obtained from all enrolled patients and/or their parents/legal guardians in accordance with the Helsinki Declaration. The study protocol was approved by the National Medical Ethics Committee. Patients who had completed orthodontic treatment with FOA in both jaws and exhibited at least one WSL on a buccal tooth surface, coded 1 (a visible carious opacity or discoloration seen after 5 s of air drying) or 2 (distinct visual change in enamel seen in wet) according to International Caries Detection and Assessment System (ICDAS), (Ismail et al., 2007) and had given consent to participate in the study, were eligible for enrollment. Exclusion criteria were (a) developmental defects of dental hard tissue, (b) caries risk increasing systemic disease or drug use, (c) debonding of the FOA due to inadequate oral hygiene, and (d) fluoride aversion.

Before bonding, all included patients had participated in oral hygiene coaching lessons to achieve a plaque index of less than 10%, which was a standard protocol at DO. During active orthodontic treatment, patients were referred to an appointed dentist to be motivated on oral hygiene, for professional teeth cleaning to be performed and FV applied half‐yearly. In all patients, bonding of the FOA was performed on acid‐etched surfaces (36% phosphoric acid) using conventional light‐cure adhesive (Enlight Light Cure; SDS, Ormco, CA, USA). The same bracket system was used in all patients (Discovery metal brackets, System Roth 22 (22 × 30 slot size; Dentaurum, Springen, Germany).

### 2.2 Study design

Debonding of FOA was performed at DO, and patients were sent to DPPD on the same day for a thorough dental examination and LF measurements. Immediately after dental examination and LF measurements, eligible patients were sent back to DO, where they were randomized into test and control groups by the examiner at DO according to a randomization table. FV or placebo was applied at DO at the same time. The randomization table with a block of 5 was prepared by the examiner at DO before the first patient was included using SPSS 16 statistical software (Statistical Package for the Social Sciences Inc., Chicago, IL). The allocation was concealed from patients and the examiners at DPPD, who assessed clinical and LF status, thereby ensuring the double‐blindness of the study.

Dental examinations and LF measurements at DPPD and fluoride/placebo applications at DO were repeated in the same manner 14 days, 4 months, and 6 months after debonding of FOA. The rationale for this interval was to adapt the fluoride applications to retention check‐ups, so the patients would not be burdened additionally following our study design.

### 2.3 Visual inspection and LF measurement

The two examiners at DPPD performed the dental examination on wet and dried debris‐free teeth, under artificial light, using a dental mirror and ball‐ended probe. WSLs, identified with visual inspection on mesiobuccal, buccal, or distobuccal tooth surfaces, were recorded. An ICDAS code was assigned to each WSL and the initial dental status was digitally photographed (Canon EOS 300, EF‐S 60 mm f/2.8 Macro USM lens).

WSLs that extended from the central buccal surface to the mesial or distal buccal surface were marked as buccal. WSLs that were coded 3 (localized enamel breakdown due to caries with no visible dentin or underlying shadow) or higher (from underlying dark shadow from dentin with or without enamel breakdown to an extensive distinct cavity with visible dentin) according to ICDAS on check‐up visits were excluded from further investigation and were treated appropriately.

A calibrated DIAGNOdent (KaVo Dental GmbH, Biberach, Germany) flat tip was used to assess the maximum LF value on each WSL coded 1 or 2 after the 0 LF value was determined for each tooth individually on a healthy surface.

On check‐up visits, LF measurements were performed on all areas that had been marked on initial photographs as WSLs, whether they were still existing WSLs or they had changed to WSL‐free areas. LF measurements were performed by the same examiners at DPPD, who also performed the visual inspection.

### 2.4 Intervention

All patients were instructed on proper tooth brushing twice daily, using toothpaste with a fluoride concentration of 1450 ppm. The same instructions were advocated again 14 days, 4 months, and 6 months after debonding. Four orthodontists who treated patients and performed debonding were instructed on how to apply varnish/placebo on all teeth in the mouth. In the test group, 0.1% FV (Fluor Protector; Ivoclar Vivadent, Schaan, Lichtenstein) was applied according to the manufacturer's instructions: teeth were dried with compressed air and cotton rolls. Varnish was applied on all teeth surfaces using a mini‐brush and it was allowed to dry for 1 min. Patients were advised not to eat or drink for 1 h after varnish application. In the control group, water, stored in thoroughly cleaned Fluor Protector bottles, was applied in the same manner as FV and the same instructions were given to the patients. Patients were blinded for the fluid applied.

### 2.5 Statistical considerations

We assumed that the clinically relevant difference between study groups in the mean number of WSLs would be four, with a standard deviation of 4.5. It was calculated that 21 patients in each group should be included to meet the above assumptions, if the *α* and *β* values were set at .05 and .2, respectively.

The intraclass correlation coefficient (ICC) was calculated for the two examiners at DPPD performing a visual inspection and LF readings on 10 patients two times in 1‐week interval. ICC estimates and their 95% confidence intervals (CIs) were calculated based on a two‐way mixed‐effects model.

There were two main outcomes. The first was the mean number of WSLs per patient identified solely by visual inspection at four‐time points in the test and control groups. The second was the mean LF value of visually identified WSLs per patient at four‐time points in the test and control groups. Differences between the test and the control group in the number of WSLs that transited to a different code or stayed the same during the study were analyzed with the *t*‐test. Differences between the two groups in the mean number of WSLs and the mean LF values of WSLs per patient at different time points were statistically analyzed with a mixed‐design analysis of variance at a level of significance *α =* .05.

In the second step, the effect of the duration of orthodontic therapy on the mean number of WSLs and the mean LF value per patient was investigated. This variable was included in the model as a covariate. The Bonferroni multiple‐comparison test was used to compare the variable at different time points.

## 3 RESULTS

**Figure 1:**
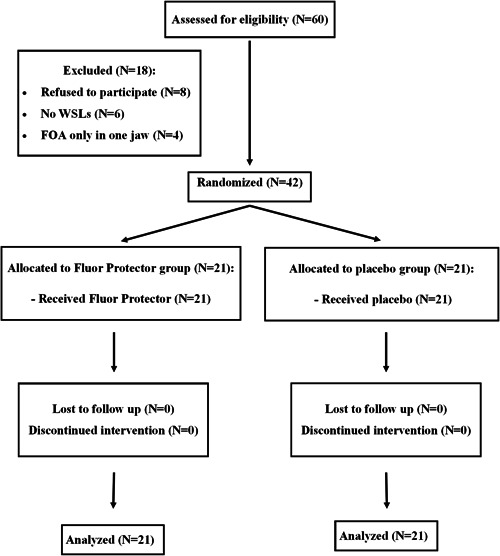
The flow diagram of enrollment, intervention allocation, follow‐up and analysis.

60 patients, who finished orthodontic therapy with FOA, were consecutively examined and were invited to participate in the study. Forty‐two of them met all the inclusion criteria. Of the 18 patients who were not included, eight patients did not consent to participate in the study (four gave the time constraint as a reason, four gave no reason), no WSLs were found in six patients, and four only had FOA in one jaw (Figure 1).

The test group consisted of 21 patients, with a female/male ratio of 16/5 and a mean age of 17.4 ± 2.8 (range: 13–26) years. Corresponding numbers in the control group, which consisted of 21 patients, were 12/9 and 17.9 ± 3.6 (range: 14–29). The mean duration of orthodontic therapy with FOA in the test and control groups was 39.6 ± 18.7 and 39.9 ± 24.6 months, respectively.

### 3.1 Visual inspection

The interexaminer ICC for visual inspection was 0.70 (95% CI: 0.68–0.73). Intraexaminer ICC for the first and second examiner was 0.72 (95% CI: 0.70–0.74) and 0.75 (95% CI: 0.72–0.77), respectively.

**Figure 2:**
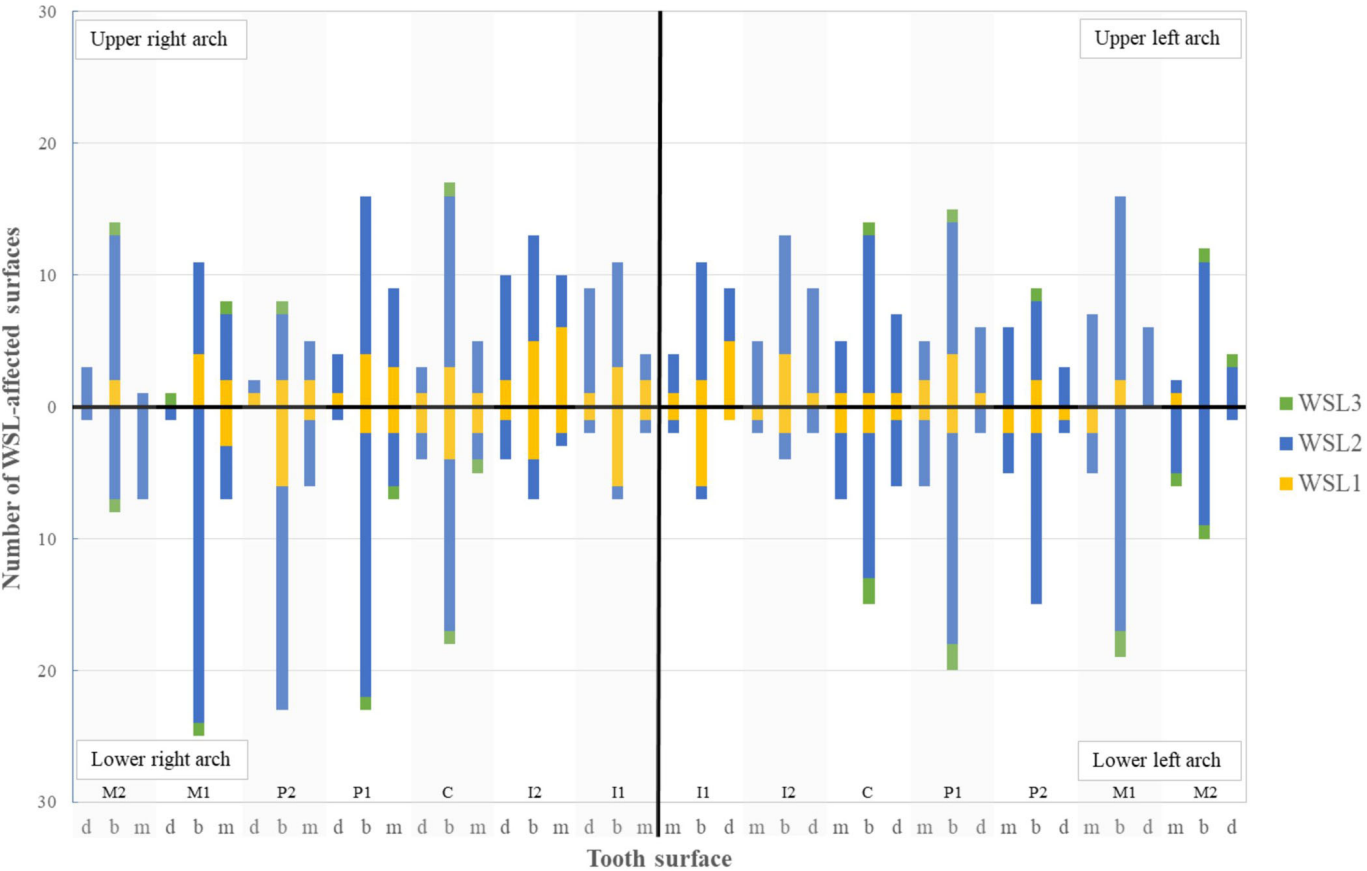
The distribution of white spot lesions (WSLs) per tooth type and tooth site (M2, M1 = second and first molar, P2, P1 = second and first premolar, C = canine, I2, I1 = second and first incisor; d = distal, b = buccal, m = mesial. WSL1 = ICDAS 1, WSL2 = ICDAS 2, WSL3 = ICDAS 3 or more)

Altogether, 1148 teeth were examined for the presence of WSLs. 606 buccal, mesiobuccal or distobuccal surfaces were identified with WSLs coded 1 or 2 according to ICDAS. Twenty‐four surfaces were coded 3 or more and were excluded from further analysis. More than three quarters (77.4%) of WSLs were coded 2. WSLs were most often noted on buccal surfaces of first molars, first premolars, and canines in the upper and lower jaw. Upper incisors were also among the most affected teeth, but WSLs were distributed more evenly on mesial, buccal, and distal surfaces, while the least affected teeth were lower incisors. The distribution of WSLs per tooth type and per tooth site is presented in Figure 2.

At the final examination, it was noted that 216 WSLs (35.6%) were not visually identifiable. However, 318 WSLs (52.5%) did not change their code over a period of 6 months. The numbers of WSLs that retained the same code or changed to a lower or higher code during the study period were not statistically significantly different between the study groups (Table 1).

**Table 1:** Numbers and percentage (%) of surfaces coded 1 or 2 at the first examination that retained the same code (1 → 1, 2 → 2), transited to lower (1 → 0, 2 → 0, 2 → 1) or higher (1 → 2, 1 → 3, 2 → 3) visual code 6 months after debonding, according to allocation to study groups

	1 → 0	1 → 1	1 → 2	1 → 3	2 → 0	2 → 1	2 → 2	2 → 3
	*p* = .542	*p* = .134	*p* = .956		*p* = .628	*p* = .373	*p* = .598	*p* = .130
Test group (*N* = 293)	45 (15.4)	6 (2.0)	20 (6.8)	2 (0.7)	65 (22.2)	8 (2.7)	143 (48.4)	4 (1.4)
Control group (*N* = 313)	38 (12.1)	4 (1.3)	23 (7.3)	0 (0)	68 (21.7)	12 (3.8)	165 (52.7)	3 (1.0)
Total (*N* = 606)	83 (13.7)	10 (1.7)	43 (7.1)	2 (0.3)	133 (21.9)	20 (3.3)	308 (50.8)	7 (1.2)

**Figure 3:**
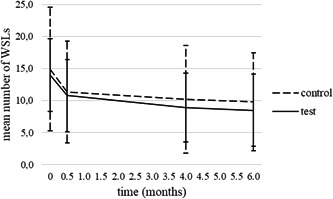
Numbers of WSLs per patient in study groups at each examination (mean ± SD). WSL, white spot lesion

The mean number of WSLs per patient decreased from 14.4 ± 7.8 to 9.1 ± 6.7 in 6 months. In the test and control groups, it dropped from 14.0 ± 5.7 to 8.5 ± 5.6 and from 14.9 ± 9.6 to 9.8 ± 7.6, respectively (Figure 3).

The results of the mixed‐design analysis of variance showed that there was no statistically significant difference in the mean number of WSLs per patient between study groups at any time point (*p* = .749). The mean number of WSLs per patient was not affected by the duration of orthodontic treatment (*p* = .605).

Without taking covariates into account, the results of a mixed‐design analysis of variance showed that there was a significant difference in the mean number of WSLs per patient between time points. The Bonferroni multiple‐comparison test showed that there was a significant drop in the mean number of WSLs per patient between the first examination and the check‐up after 14 days and all the following check‐ups (*p* < .001). However, the drop was not significant between the check‐ups at 4 and 6 months (*p* = 1).

### 3.2 LF readings

Interexaminer ICC for LF readings was 0.95 (95% CI: 0.94–0.95). Intraexaminer ICC for first and second examiner was 0.84 (CI: 0.83–0.85) and 0.74 (CI: 0.72–0.77), respectively.

**Figure 4:**
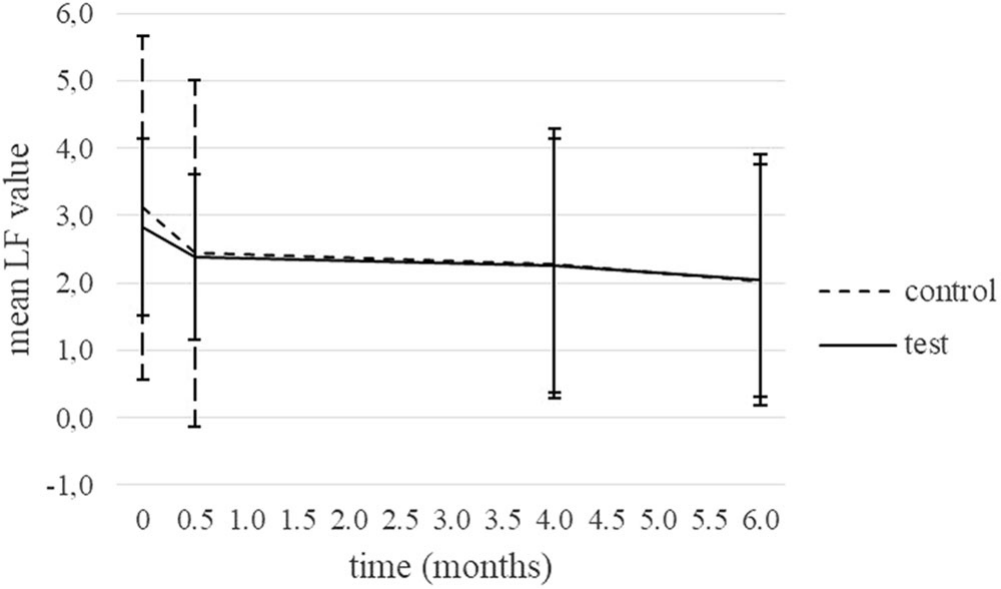
LF values of WSLs per patient in study groups at each examination (mean ± SD). LF, laser fluorescence; WSL, white spot lesion

The results of the visual inspection were confirmed by LF measurements since LF values also decreased in time: At the first examination, the mean LF value was 3.0 ± 2.0 and after 6 months it was 2.0 ± 1.8. The mean LF value per patient decreased from 2.8 ± 1.3 to 2.0 ± 1.9 in the test group and from 3.1 ± 2.6 to 2.0 ± 1.7 in the control group (Figure 4).

LF values changed to approximately the same extent in both groups since according to the mixed‐design analysis of variance there were no significant differences in mean LF values per patient between the two groups at any time point. The duration of treatment with the FAO was in interaction with the mean LF value per patient (*p* = .01).

**Figure 5:**
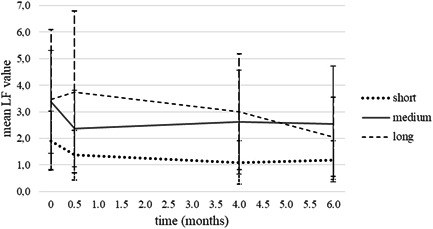
LF values of WSLs per patient at different examinations in groups of patients with short, medium, and long duration orthodontic treatment (mean ± SD). LF, laser fluorescence; WSL, white spot lesion

Further analysis showed that in patients who were treated with FOA for more than 48 months (long duration orthodontic treatment group; including five patients from the test and five patients from the control group), mean LF values at different time points decreased differently than in patients who were treated with FOA for 24 months or less (short duration orthodontic treatment group, including four patients from the test and eight patients from the control group) or in patients who wore FOA for 25–48 months (medium duration orthodontic treatment group, including 12 patients from the test and eight from the control group) (Figure 5). In the long‐ and medium‐duration orthodontic treatment groups, the mean LF values per patient were higher at first examination and 6 months after debonding than in the short orthodontic treatment duration group, but the differences between the groups were not statistically significant. However, 14 days after debonding there were significant differences between long‐ and short‐duration orthodontic treatment groups (*p* = .013), with an increase in the mean LF values per patient in the long duration orthodontic treatment group. Four months after debonding there were significant differences between long‐ and short‐duration orthodontic treatment groups (*p* = .047), and the difference was approaching significant between medium‐ and short‐duration treatment groups (*p* = .072).

## 4 DISCUSSION

WSLs are disturbing side effects of treatment with FOA and are reported to be long‐lasting in most cases (Mattousch et al., 2007). In the present study, 64.4% of WSLs coded 1 and 2 according to ICDAS were still present after the 6‐month period of observation. As was shown by Shungin et al. (2010) a certain percentage of WSLs remain over longer periods of time, even 12 years after debonding. A significant drop in the number of WSLs was seen in the first 14 days after the removal of FOA and the drop was less obvious after 4 and 6 months. The results obtained by visual inspection were confirmed by the results obtained by measurement of LF on WSLs as the curves in Figures 1 and 2 show the same trend over 6 months. In our study, 32.7% of WSLs coded 2 regressed to codes 1 or 0, which is consistent with the findings of Beerens et al. (2015) who noted almost the same percentage (30.3%) of improved postorthodontic WSLs coded 2 according to ICDAS over a 1‐year period.

The present study was undertaken to evaluate the effect of 0.1% FV Fluor Protector on the regression of WSLs. The results showed that in our study group, 0.1% FV application did not produce a significantly better effect on WSL regression, assessed by visual scoring and LF, in comparison to advocated oral hygiene including the use of fluoride toothpaste alone, so our hypothesis was rejected and the null hypothesis confirmed. Secondly, since the WSL regression course was similar in both study groups, putative premature hardening of the lesion surface due to the 0.1% FV application was not considered to be a cause of concern.

We hypothesize that improved oral hygiene, including the abrasive effect of tooth brushing (Artun & Thylstrup, 1986) with fluoride toothpaste after removal of retentive orthodontic elements, and the effect of saliva, (Ogaard & Ten Bosch, 1994) were the main mechanisms affecting the reduction of the number and LF values of WSLs in our study. It has been reported that 5% FV Duraphat contributed to a greater decrease in DIAGNOdent Pen readings on WSLs than saline (Du et al., 2012). Moreover, it induced a greater decrease of WSL volume than toothpaste or fluoride film, measured with quantitative light‐induced fluorescence (He et al., 2016). However, the results were not supported by clinical examination. Consistent with our study results, Huang et al. (2013) reported no beneficial effect of 5% FV compared to standard oral hygiene and the use of fluoridated toothpaste, evaluated by assessing WSL improvement on photographed teeth.

For orthodontic patients, the application of FV is beneficial during treatment with FOA to prevent the development of WSLs (Benson et al., 2013). However, the evidence is conflicting for the use of FV as a treatment for WSLs after debonding. A limitation in studying the effect of FV, also in our study, is the concomitant use of fluoridated toothpaste; however, for ethical reasons, the use of toothpaste cannot be omitted.

Even though no significant interaction between WSL number and treatment duration was established in our study, an interaction was shown between LF values of WSL and the duration of orthodontic therapy. It was noted that the values increased 14 days postdebonding in the group of patients who were treated with FOA for more than 48 months. A possible explanation for this increase may be in abrasion of the lesion surface exposed to the more demineralized lesion body after establishing conditions for proper tooth‐brushing. Laitala et al. (2017) showed that occlusal initial lesions with LF less than 20 improved better as lesions with LF more than 30 after a month of targeted tooth brushing, which could also be seen in our study at the beginning of the observation period. It has been shown that long orthodontic treatment (>36 months) is a risk factor for WSL development and more severe WSLs (Julien et al., 2013) and that higher LF values of WSLs were associated with deeper carious lesions (Shi et al., 2011). In the group of patients who were treated for more than 48 months, the drop of LF values of WSLs over the observation period was greater than in the groups of patients who were treated with FOA for less than 24 months or patients who were treated with FOA from 25 to 48 months. If we assume that higher LF values mean more demineralized WSLs (Diniz et al., 2015) in patients who were treated for more than 48 months, our results seem to be consistent with the results of the study performed by van der Veen et al. (2007), in which better remineralization was shown in deeper lesions than in more superficial enamel lesions, using quantitative light‐induced fluorescence. To obtain more valid data for exploring the interaction between LF values of WSLs on smooth surfaces and the duration of orthodontic therapy in our study, more time points with more consistent interims would probably be beneficial. Since this was not our primary outcome, but an incidental finding, we aim to perform further research with the limitations of this study taken into consideration.

## 5 CONCLUSION

WSLs are a common unwanted side effect of treatment with a FOA. FV can be used to facilitate WSL's regression.

In the present study, the decrease in the number and LF value of WSL was significant over 6 months, but the effect of 0.1% FV was not confirmed. LF measurements indicated different modes of WSL regression regarding orthodontic therapy duration.

Further studies are needed to establish an efficient treatment protocol for WSLs in postorthodontic patients. Adaptation according to the duration of orthodontic therapy may prove to be necessary.

## AUTHOR CONTRIBUTIONS

Tanja Tomaževič performed clinical evaluations, collected data, and wrote the manuscript; Martina Drevenšek consulted for statistical evaluation, contributed substantially to the discussion, and proofread the manuscript; Rok Kosem conceived the idea and experimental design and proofread the manuscript.

## CONFLICTS OF INTEREST

The authors declare no conflicts of interest.

## Data Availability

The data that support the findings of this study are available from the corresponding author upon reasonable request.
